# Supplementary material for: From sequence to enzyme mechanism using multi-label machine learning
Source: BMC Bioinformatics. 2014 May 19;15:150. doi: 10.1186/1471-2105-15-150 (PMC4229970; doi:10.1186/1471-2105-15-150)
Supplement: Additional file 2 — Java code of ml2db. Additional file ml2db_code.tar.gz contains the Java source code to run the multi-label machine learning experiments and save the results to database. The code’s Javadoc is included. [file 1471-2105-15-150-S2.zip › additional file 2/ml2db/ml2db/doc/mulan/evaluation/loss/class-use/BipartitionLossFunction.html]

Uses of Interface mulan.evaluation.loss.BipartitionLossFunction


JavaScript is disabled on your browser.


- Overview
- Package
- Class
- Use
- Tree
- Deprecated
- Index
- Help

- Prev
- Next

- Frames
- No Frames

- All Classes

## Uses of Interface mulan.evaluation.loss.BipartitionLossFunction

- Packages that use BipartitionLossFunction

  | Package | Description |
  | --- | --- |
  |  |  |
  | --- | --- |
  | mulan.evaluation.loss |  |
  | mulan.evaluation.measure |  |
- - ### Uses of BipartitionLossFunction in mulan.evaluation.loss

    Classes in mulan.evaluation.loss that implement BipartitionLossFunction

    | Modifier and Type | Class and Description |
    | --- | --- |
    |  |  |
    | --- | --- |
    | `class` | `BipartitionLossFunctionBase` Base class for bipartition loss functions |
    | `class` | `HammingLoss` Implementation of the hamming loss function. |
    | `class` | `HierarchicalLoss` Implementation of the hierarchical loss function. |
  - ### Uses of BipartitionLossFunction in mulan.evaluation.measure

    Constructors in mulan.evaluation.measure with parameters of type BipartitionLossFunction

    Constructor and Description

    `LossBasedBipartitionMeasureBase(BipartitionLossFunction aLoss)`

    Creates a loss-based bipartition measure

- Overview
- Package
- Class
- Use
- Tree
- Deprecated
- Index
- Help

- Prev
- Next

- Frames
- No Frames

- All Classes
